# MRI for biology‐guided radiation therapy: Are we there yet? A summary of the 2024 ISMRM member‐initiated session

**DOI:** 10.1002/mrm.30616

**Published:** 2025-06-24

**Authors:** Sirisha Tadimalla, Jie Deng, Peter B. Barker, Hyunsuk Shim, Stefan Reinsberg, Chenyang Liu, Jing Cai, Jonathan Goodwin, Leith Rankine, Yu‐feng Wang, Petra van Houdt, Ralph P. Mason, Zhaoyang Fan

**Affiliations:** ^1^ Institute of Medical Physics, The University of Sydney Sydney New South Wales Australia; ^2^ Department of Radiation Oncology University of Texas Southwestern Medical Center Dallas Texas USA; ^3^ The Russell H. Morgan Department of Radiology and Radiological Science Johns Hopkins University School of Medicine Baltimore Maryland USA; ^4^ Kennedy Krieger Institute Baltimore Maryland USA; ^5^ Department of Radiation Oncology Emory University Atlanta Georgia USA; ^6^ Department of Physics and Astronomy University of British Columbia Vancouver British Columbia Canada; ^7^ Department of Health Technology and Informatics Hong Kong Polytechnic University Hong Kong China; ^8^ Radiation Oncology Department Calvary Mater Newcastle Newcastle New South Wales Australia; ^9^ School of Information and Physical Sciences College of Engineering, Science and Environment, The University of Newcastle Newcastle New South Wales Australia; ^10^ Department of Radiation Oncology University of North Carolina Chapel Hill North Carolina USA; ^11^ Department of Radiation Oncology The Netherlands Cancer Institute Amsterdam The Netherlands; ^12^ Prognostic Imaging Research Laboratory, Department of Radiology UT Southwestern Medical Center Dallas Texas USA; ^13^ Departments of Radiology and Radiation Oncology, Keck School of Medicine University of Southern California Los Angeles California USA

## Abstract

At the 2024 ISMRM Annual Meeting in Singapore, a member‐initiated session on MRI for biology‐guided radiation therapy (RT), endorsed by the ISMRM MR in RT Study Group, was successfully organized. The session convened a diverse group of global experts in quantitative MRI for RT, who presented the latest research on the technical development and clinical translation of various quantitative MRI techniques for biology‐guided RT planning and delivery. The session highlighted clinical needs and a variety of MRI techniques, including MR spectroscopic imaging, oxygen‐enhanced MRI, four‐dimensional MR fingerprinting, dynamic contrast‐enhanced MRI, and ^129^Xe MRI. Additionally, technical aspects and challenges for clinical translation of quantitative MRI into biology‐guided RT were presented, both in the context of RT planning and adaptation. This article summarizes the progress made in this emerging field, identifies key challenges that need to be addressed, and outlines areas for future research. These insights are crucial for the integration of quantitative MRI techniques into RT clinical practice, ultimately aiming to improve patient outcomes through more personalized RT approaches.

## 1 INTRODUCTION

The potential for the use of quantitative MRI (qMRI) techniques to obtain microstructural, metabolic, and physiological information from tumors and normal tissues has long been recognized.^1^ With advancements in hardware and software technologies, there is a growing interest in the opportunities presented by qMRI to personalize radiation therapy (RT).^2^ Biology‐guided radiation therapy is an emerging RT paradigm in which tissue biological characteristics directly inform the planning and delivery of radiation treatment. For instance, qMRI can be used to improve the accuracy in the identification of tumor margins to optimize treatment planning and deliver effective radiation dose to the tumor, while minimizing off‐target damage and sparing function of normal tissues. Additional opportunities are provided by qMRI of tissue characteristics to predict radiation sensitivity or provide early indication of radiation treatment response. Notably, hypoxia is recognized as a key driver of radiation resistance; thus, there are potential opportunities to use qMRI to identify hypoxic regions to enable targeted dose modulation.^3^ Similarly, other qMRI parameters such as the apparent diffusion coefficient (ADC) derived from diffusion weighted imaging (DWI) and relative cerebral blood volume derived from dynamic‐susceptibility‐contrast perfusion MRI may be used to delineate necrosis and assess treatment efficacy.^4^, ^5^ With recently introduced technologies for real‐time volume tracking in situ, the prospect of using qMRI for adaptive RT for more effective targeting of RT delivery is becoming increasingly feasible.

At the 2024 ISMRM annual meeting in Singapore, a member‐initiated session on MRI for biology‐guided RT was successfully organized. Eight experts were invited to present their insights and research on the development and clinical translation of various qMRI techniques. The experts were selected based on recent publications, ongoing clinical trials on MRI for biology‐guided RT, and availability to attend the conference. The following review provides a summary of the scientific content covered by the session, highlighting the progress made and the future directions in this promising field.

### 1.1 Prelude

#### 1.1.1 Jie Deng

**FIGURE 1:**
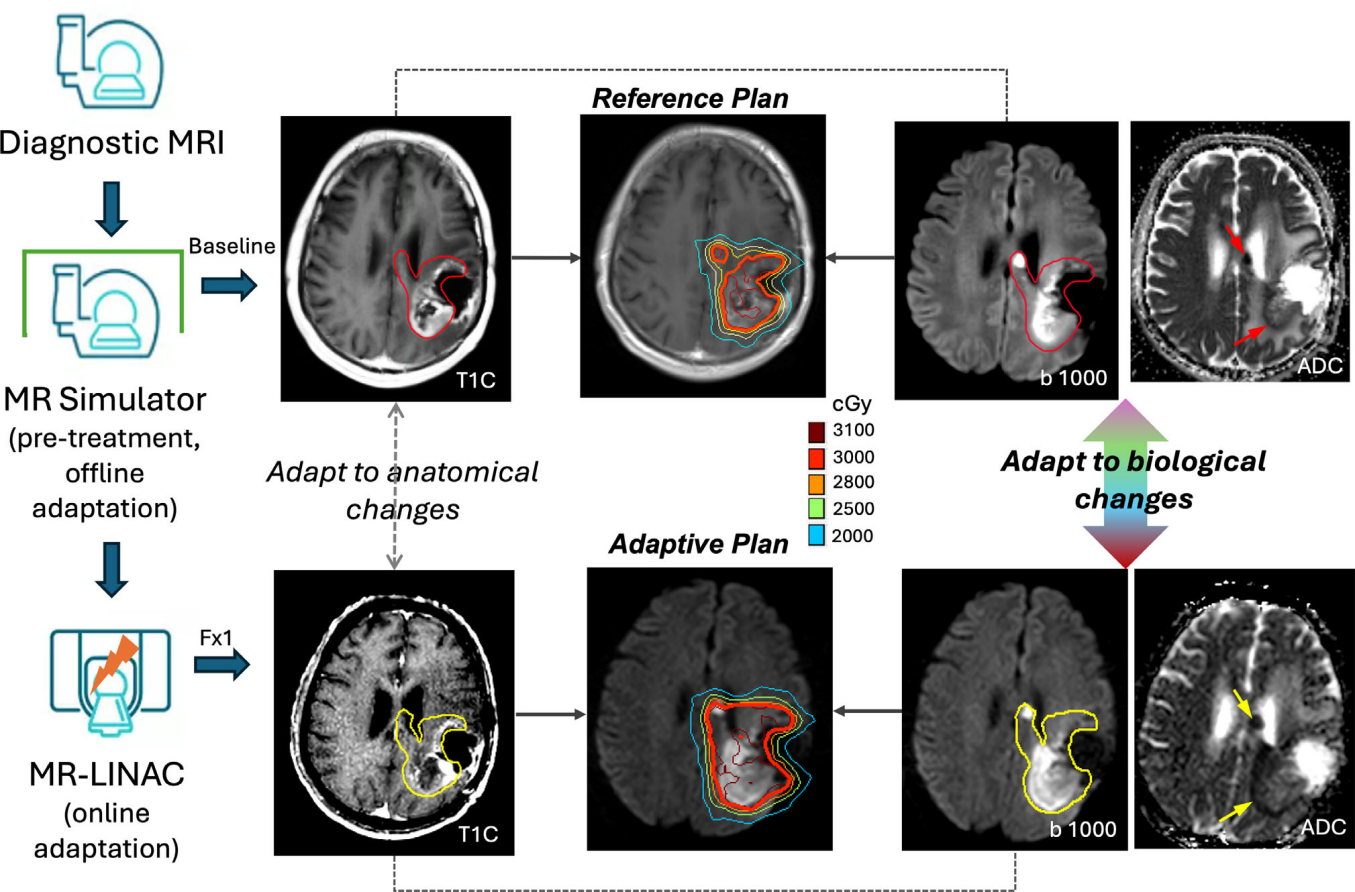
Biology‐guided radiation therapy (RT) workflow during MRI simulation and treatment adaptation. In this example, post‐contrast‐enhanced T_1_‐weighted (T1C) images, diffusion‐weighted imaging at *b*‐value of 1000 s/mm^2^, and corresponding apparent diffusion coefficient (ADC) maps are used jointly to delineate the target. For treatment adaptation, T1C images are first used for image fusion and anatomic adaptation based on positional and shape changes, followed by biological adaptation based on the evaluation of diffusion‐restricted areas.

MRI has been used increasingly in image‐guided RT to enhance treatment precision and outcomes by exploiting its superior soft‐tissue contrast and versatile information. For some cancer sites such as brain, head/neck, abdomen, and pelvis, MRI simulation (MR‐Sim) is a standard procedure for treatment planning, where baseline MR images are acquired on a whole‐body scanner with devices for precise patient positioning, replicating the radiation treatment setup.^6^ Traditionally, midtreatment MRI, acquired on an MR‐Sim or diagnostic scanner, allows for offline adaptation of treatment plans based on anatomical changes such as tumor size, shape, and position. However, the introduction of hybrid MR‐linear accelerator (MRI‐LINAC) systems has transformed RT by integrating MRI directly into treatment delivery.^7^ These systems provide daily MRI to enable online adaptation of treatment plans to accommodate daily variations in tumor and organs at risk (OARs) positions and shapes, ensuring optimal target volume, which is a significant step toward personalized RT. Beyond its role in anatomical imaging for tumor and OAR delineation, MRI provides unique capabilities for assessing the tumor microenvironment and changes in biological function of tumors and OARs. Biology‐guided adaptation using MRI can potentially be used to tailor RT to individual patient responses, enhance treatment efficacy, and reduce toxicity. Figure 1 illustrates a biology‐guided RT workflow throughout the procedures of MRI simulation and treatment, integrating DWI for contour adjustment and plan adaptation to biological changes, either offline using an MR‐Sim or online on the MRI‐LINAC.

Understanding radiobiology mechanisms and designing pertinent dosing strategies are essential for augmenting RT effectiveness. From the radiobiology perspective, tumor cells undergo various pathways of cell death following RT—from mitosis to apoptosis and necrosis—indicative of treatment outcomes. Radiation also influences tumor vasculature, potentially restructuring chaotic blood vessel networks within tumors. Moreover, tumor hypoxia affects the response to RT through resistance, and monitoring the spatial–temporal dynamics of hypoxia throughout RT could be crucial for tumor control. These biological responses underscore the importance of integrating radiobiological insights into RT planning and execution to optimize therapeutic outcomes. In addition, dosing strategies in RT are evolving from conventional fractionated schedules to hypo‐fractionated approaches, enabled by advances in image guidance. These strategies deliver higher doses per fraction with fewer fractions over a short period, thus enhancing tumor control while minimizing damage to healthy tissues.^8^ More recently, ultra‐hypo‐fractionated RT was introduced with reduced toxicity and long intervals between fractions to facilitate meaningful adaptation to patient‐specific biomarkers, as well as foster adaptive immunity.^9^

Looking ahead, the future of adaptive RT will benefit from integrating biological information obtained through MRI. Continuous feedback on biological changes in tumors and OARs will refine treatment strategies, moving toward truly personalized RT across clinical settings. Achieving this vision requires collaboration among MRI scientists, medical physicists, and clinicians to develop and validate imaging biomarkers that reliably reflect biological responses to RT. Bridging this gap will expedite the translation of advanced MRI techniques from preclinical studies to clinical practice, ultimately improving patient outcomes.

In conclusion, MRI's role in biology‐guided RT is rapidly evolving, driven by advancements in imaging technology and our understanding of radiobiology. By leveraging MRI capabilities for both anatomical and functional imaging in individual patients, we are progressing toward realizing the full potential of RT in cancer treatment.

### 1.2 Spectroscopic MRI‐guided RT in brain tumors

#### 1.2.1 Peter Barker, Hyunsuk Shim

Glioblastoma (GBM) is the most aggressive form of brain cancer and accounts for 49% of all malignant tumors. There are over 12 000 new cases per year in the United States.^10^ Despite aggressive treatment with surgery followed by chemoradiation therapy, median survival remains a little over a year. One reason for treatment failure is that conventional MRI scans (e.g., postcontrast T_1_‐weighted MRI) used for radiation treatment planning may not capture the entire extent of tumor, particularly because of the infiltrative nature of GBM. Consequently, treatment plans often include a 5–10‐mm margin around MRI‐defined tumor regions. However, this does not guarantee successful treatment and may also damage the surrounding normal brain tissues.

Proton MR spectroscopic imaging has been investigated in brain RT^11^ and may visualize regions of invasive tumor better than conventional MRI, distinguishing it from peritumoral edema. Gurbani et al. have developed a technique called spectroscopic MRI (sMRI), which combines high‐resolution whole‐brain metabolic imaging with methods for postprocessing, visualization, and treatment planning.^12^ This provides the basis for the integration of metabolic imaging into the clinical workflow for brain tumor management.

A multi‐institutional pilot clinical trial of sMRI‐guided radiation dose escalation for newly diagnosed GBM^13^ (NCT03137888) demonstrated the safety and feasibility of dose escalation to 75 Gy. sMRI was performed at 3 T with whole‐brain coverage, and an isotropic, nominal spatial resolution of about 108 mm^3^ (echo planar spectroscopy imaging, GRAPPA [generalized auto‐calibrating partially parallel acquisition] accelerated, repetition time [TR]/echo time [TE] = 1551/50 ms, scan time = 15 min). In addition to the standard tumor volumes delineated based on conventional T_2_‐FLAIR and contrast‐enhanced T_1_‐weighted imaging, an additional high‐risk volume based on residual contrast‐enhanced tumor and the Cho/NAA ratio (on sMRI) at least twice that of normal tissue was determined and treated with 75 Gy. Thirty newly diagnosed GBM patients were treated in the study. The median age was 59 years. Thirty percent were O^6^‐methylguanine‐DNA methyltransferase–promoter hypermethylated, and 7% harbored isocitrate dehydrogenase 1 mutation—both of which are associated with improved treatment outcome. The median overall survival was 23 months,^13^ compared with 16 months in historical controls.^14^, ^15^ This regimen was well‐tolerated, with 70% of Grade 3 or greater toxicity ascribed to Temozolomide, 23% of which occurred at least 1 year after RT.

These results are encouraging and warrant further randomized studies in larger cohorts of subjects. Compared with other metabolic imaging techniques (e.g., amino acid positron emission tomography [PET]), sMRI is relatively inexpensive, noninvasive, and repeatable. However, improvements are still needed to accelerate data acquisition and metabolic image reconstruction for timely interpretation and therapy planning. Due to its sensitivity to B_0_‐field inhomogeneities, advanced software‐based or hardware‐based field shimming is clinically warranted. Multivendor implementation and standardization are also required for widespread dissemination.

### 1.3 Oxygen‐enhanced MRI in RT

#### 1.3.1 Stefan Reinsberg, Ralph Mason

It has long been appreciated that hypoxia influences radiation response. Hypoxic tumors are associated with a more aggressive phenotype and generally respond less well to radiation in terms of biological relapse and overall survival.^3^ Numerous studies examining tumors at various disease sites have revealed hypoxic tumors based on measurements with invasive needle electrodes, such as in cervical cancer.^16^ The ARCON clinical trial examined head and neck tumors based on quantification of pimonidazole in biopsies.^17^ Enhanced response was shown by application of the modified treatment procedure with accelerated radiation, breathing carbogen and administering nicotinamide. However, there is clearly a need for noninvasive procedures to identify tumor hypoxia. A recent study used the bio‐reductively activated hypoxia marker F‐misonidazole and PET to identify hypoxic tumors.^18^ It was then shown that de‐escalated chemoradiotherapy was as effective in patients with oxic tumors as standard therapy in hypoxic tumors. This suggests that there may be exceptional opportunities for dose reduction–mitigating side effects for patients with well oxygenated tumors without compromising the ultimate radiation response.

Hypoxia may be related to tumor perfusion, and procedures such as DCE‐MRI have been related to tumor hypoxia.^19^ A more direct assessment of tumor oxygenation may be derived from an oxygen gas breathing challenge accompanied by oxygen‐enhanced (OE) MRI such as blood oxygen–level dependent (BOLD) and tissue oxygen–level dependent measurements.^20^, ^21^ Specifically, R_2_* (BOLD signal) is sensitive to deoxyhemoglobin; thus, conversion of the deoxy‐hemoglobin to oxy‐hemoglobin accompanying a hypoxic gas breathing challenge gives a clear indication of oxygen delivery to tumors.^22^ However, the magnitude of response can be highly variable depending on the actual pO_2_.^23^ Meanwhile, the spin lattice relaxation rate (R_1_) of tissue is directly sensitive to pO_2_. Several research groups have explored the utility of R_1_ (or T_1_‐weighted signal) with MRI to assess preclinical tumors in animals. In some cases, a correlation has been reported between R_1_ and R_2_*, but this does not appear to be universal.^24^ Arai et al. reported that rat tumors exhibiting no response to an oxygen gas breathing challenge before irradiation had much poorer tumor control than those tumors that did respond (i.e., those that did not exhibit hypoxia).^25^ Moreover, a radiation boost to those tumors assessed to be hypoxic overcame the radiation resistance. O'Connor et al. have shown the need for including DCE‐MRI with oxygen‐enhanced T_1_‐sensitive MRI in some tumor types, to differentiate hypoxia from necrotic tissue and obtain stronger correlations with histologically defined hypoxia and necrosis.^21^ As an alternative, correlated BOLD and tissue oxygen–level dependent MRI can establish whether a response is consistent with oxygen delivery.^22^ R_1_ responses in numerous preclinical tumor lines have been examined, verifying effective identification of hypoxic extent and the ability to modify hypoxia based on vascular endothelial growth factor ablation.^26^, ^27^ They applied several cycling oxygen challenges to enhance identification of response based on independent component analysis that overcomes the limitations of signal‐to‐noise ratio.

Several studies have now indicated the feasibility of making oxygen‐sensitive MRI measurements in human patients at multiple disease sites.^28^, ^29^, ^30^ The R_1_ response is indeed quite small; thus, it is crucial to minimize potential artifacts such as motion. A recent study indicated effective oxygen‐sensitive MRI measurements in a cohort of 24 head and neck cancer patients.^31^ They examined reproducibility of measurements as well as changes accompanying RT. However, it is crucial to develop such methods at multiple institutions to validate the methodology, the repeatability, and ultimately the sensitivity and specificity for predicting radiation response. The prospective MANGO trial in glioblastoma in Australia proposes to rigorously assess correlations among R_1_, R_2_*, DCE‐MRI, and F‐misonidazole PET and is anticipated to provide a convincing foundation for future investigations.^32^ Predictive validation is required to optimize oxygen inhalation duration, define hypoxia response thresholds, and identify tumor regions for dose escalation, to establish a robust OE‐MRI prognostic biomarker. Although OE‐MRI is low‐cost and uses oxygen gas as the reporter agent, gas breathing challenges may be unsuitable for patients with respiratory conditions. Additionally, staff may be less familiar with gas‐mask fitting than intravenous cannulation. Overcoming these challenges will support widespread adoption of OE‐MRI.

### 1.4 Respiratory‐correlated four‐dimensional MR fingerprinting for liver cancer radiotherapy motion management

#### 1.4.1 Chenyang Liu, Jing Cai

Effective management of respiratory motion is crucial in liver cancer radiotherapy. Poor management may result in errors in tumor volume delineation, patient positioning, and treatment delivery, leading to unnecessary liver toxicity.^33^, ^34^ Four‐dimensional MRI (4DMRI) is an essential technique for motion management in MRI‐guided RT.^35^, ^36^, ^37^ It captures detailed organ motion information throughout the patient's breathing cycle and enables precise discrimination between tumors and healthy tissues. However, several limitations of 4DMRI have hindered its widespread application, including the inconsistency of interpatient tumor contrast, the insufficiency of temporal resolutions, and the lack of quantitative and functional information.

Magnetic resonance fingerprinting (MRF) is a qMRI technique with significant potential to overcome the limitations of 4DMRI and enable 4D biology‐guided RT. MRF allows for simultaneous measurement of multiple tissue properties (e.g., T_1_/T_2_ relaxation time, proton density, fat fraction, perfusion, diffusion) within a single scan.^38^, ^39^ The MRF‐derived quantitative tissue maps provide valuable physiological and functional information^40^, ^41^ with high repeatability and reproducibility.^42^, ^43^ Several preliminary 4DMRF methods have been developed using retrospective MRF signal binning.^44^, ^45^, ^46^, ^47^ However, to acquire sufficient MRF signals for each respiratory phase after phase binning, 10 repetitive MRF scans were used, which is a time‐consuming approach that limits its clinical applicability. Recently, motion compensation–based methods have demonstrated over 5‐fold acquisition acceleration through interphase data sharing. After phase binning, MRF signals from other phases are transformed to align with the target phase, providing sufficient data for pattern matching.

Our latest MRF study, respiratory‐correlated 4DMRF (RC‐4DMRF), was the first of its kind to validate 4DMRF on prospectively enrolled liver cancer patients.^48^ A pyramid motion compensation framework was developed in RC‐4DMRF that optimizes the interphase deformation vector fields in a coarse‐to‐fine manner, enabling accurate and quantitative 4D motion imaging of liver cancer. The developed RC‐4DMRF demonstrated excellent agreement in tumor motion measurements with cine MRI and achieved the highest reconstruction accuracy among previous 4DMRF studies. The T_1_ map produced by RC‐4DMRF offered tumor‐to‐liver contrast comparable to contrast‐enhanced MRI, notably without the use of a contrast agent. The RC‐4DMRF‐derived motion‐resolved tissue maps may serve as quantitative biomarkers, facilitating the assessment of intratumoral heterogeneity and longitudinal tumor response. In addition, our recent study investigated the local T_1_ and T_2_ sensitivity by a modified Minkowski distance, indicating a shortened acquisition approach for 4DMRF reconstruction.^49^ Although RC‐4DMRF has a relatively long acquisition time (10 s per two‐dimensional slice; 3–5 minutes for a three‐dimensional [3D] volume), its contrast‐free imaging and dynamic tumor motion characterization, comparable to contrast‐enhanced MRI, may justify the added time in pretreatment workflows. For clinical translation, computational demands for fast image reconstruction must be optimized. Additionally, the utility of T_1_ and T_2_ quantification for RT guidance requires clinical validation.

In summary, RC‐4DMRF demonstrated promising performance in motion management of liver cancer in MRI‐guided and biological‐guided RT. A more comprehensive clinical study will be a valuable future direction for evaluating the benefits of RC‐4DMRF, particularly its role in internal‐target‐volume determination for treatment planning and the prognostic value of RC‐4DMRF‐derived quantitative image biomarkers for RT decision making.

### 1.5 MRI‐guided functional sparing in liver RT planning

#### 1.5.1 Jonathan Goodwin

MRI‐guided functional sparing in liver cancer RT planning represents a significant opportunity for the improved treatment of hepatocellular carcinoma (HCC). As one of the leading causes of cancer‐related deaths globally,^50^ HCC poses a substantial challenge, especially for patients with underlying liver cirrhosis, who are often not suitable candidates for liver resection, the first‐line treatment. For these patients, stereotactic body radiotherapy offers an alternative by delivering high doses of radiation in a hypo‐fractionated manner to a small volume, thus achieving improved local control and reducing the incidence of radiation‐induced liver disease (RILD).

However, the efficacy of stereotactic body radiotherapy is constrained in patients with severe liver function impairment due to an increased risk of RILD.^51^ Studies have shown that advanced cirrhotic patients exhibit lower radiation tolerance, leading to a higher incidence of liver toxicity and decreased survival rates. This necessitates the development of strategies to mitigate treatment‐related liver damage, with underlying liver function being a critical predictor of outcomes.

Traditional methods for assessing liver function in radiation therapy include clinical scoring systems such as the Child‐Pugh score, Model of End‐Stage Liver Disease score, and Albumin‐Bilirubin score. These methods provide only a global measure of liver function, lacking any spatial resolution required for precise RT planning. Nuclear medicine techniques like SPECT with hepatobiliary iminodiacetic acid offer metabolic information but suffer from low spatial resolution and technical challenges in 3D imaging.

DCE‐MRI with gadoxetic acid is emerging as a promising tool to address these limitations. This method enables the quantification of liver function at a regional and voxel level, providing detailed functional maps that can be integrated into RT planning. One approach involves acquiring baseline and contrast‐enhanced images at various phases, followed by deconvolution analysis to estimate hepatic extraction fraction (HEF) values.^52^ These HEF maps reflect liver function at a voxel level, which may facilitate precise targeting during RT. There are significant differences in HEF values across different Child‐Pugh score groups, indicating varying levels of liver function impairment. Additionally, intravoxel incoherent motion modeling of DWI has shown potential in assessing liver perfusion and distinguishing between healthy and impaired liver function, suggesting its utility in enhancing diagnostic accuracy.^53^

Application of MRI‐based functional maps in RT planning shows promising results. Retrospective assessment of DCE‐MRI‐derived HEF function‐sparing plans suggested up to a 37.5% reduction in dose to uninvolved liver volumes in some patients, compared with standard clinical plans. This reduction in radiation dose to better functioning liver is achieved while maintaining effective tumor control. Future work in this area aims to refine these techniques, including the adoption of high‐temporal‐resolution DCE‐MRI and pharmacokinetic modeling for HEF. Enhancements in image‐processing pipelines for automated HEF map generation and seamless integration into treatment planning systems are also anticipated. It is worth noting that DCE‐MRI is contraindicated in patients with severe renal insufficiency. Advances in intravoxel incoherent motion may enable it to replace DCE‐MRI, offering the advantage of contrast‐free imaging and improving feasibility for implementation on MRI‐LINACs.

In conclusion, MRI‐guided functional sparing represents an exciting area of development in liver cancer RT planning. Advanced MRI techniques available for abdominal imaging offer the potential to obtain maps of localized liver function, which may facilitate tailored treatments to individual patients, reducing the risk of RILD and improving therapeutic outcomes for those suffering from HCC.

### 1.6 ^129^Xe gas exchange MRI for functional‐avoidance RT planning and early detection of radiation‐induced lung injury

#### 1.6.1 Leith Rankine

For RT to the lung or other thoracic sites, such as esophagus, mediastinum and breast, MRI can produce potentially beneficial 3D maps of pulmonary function by imaging inhaled ^129^Xe gas. Xenon gas is soluble in tissue and in blood and follows a physiological pathway similar to inspired oxygen: filling the alveolar airspaces, dissolving into and diffusing through the alveolar membrane tissue, and then transiently bonding/interacting with the red blood cells (RBCs) within the pulmonary capillaries. ^129^Xe exhibits a chemical shift between the airspaces, membrane tissue (198 ppm), and RBCs (217 ppm). This shift in precession frequency permits the imaging and measurement of regional ventilation and gas exchange (including both alveolar “membrane uptake” and capillary “RBC transfer” interactions) in a single breath‐hold study.^54^, ^55^, ^56^, ^57^ Before the MRI, the Xenon gas must be hyperpolarized (HP) to boost the strength of the signal by a factor of 10^5^ from thermal equilibrium levels^58^ to be visible on MRI. Hyperpolarization is most often accomplished using a rubidium spin‐exchange optical pumping system.^59^

There are several ways that functional maps of the lung may be useful in thoracic RT. The first is to use pulmonary function maps to guide RT planning, which by itself is not a new concept.^60^, ^61^ This involves coregistering a map of the patient's pulmonary function with the treatment‐planning CT, then simultaneously minimizing the radiation dose to high‐functioning areas of lung and maintaining the prescribed radiation dose to the planning target volume. Until recently, functional planning has relied on either ventilation or perfusion to guide this process. However, measurements of ventilation and perfusion alone do not quantify end‐to‐end regional lung function, due to the absence of regional gas exchange information. In 2018, Rankine et al. imaged a small series of 17 human subjects, including both healthy volunteers and RT patients,^62^ using an established 15‐s breath‐hold HP ^129^Xe MRI technique.^56^ Here, the authors measured and compared the spatial distributions of regional ventilation and RBC interactions. These results demonstrated that, due to a weak‐to‐moderate correlation, ventilation may not be an effective surrogate for gas exchange in all RT patients. In 2021, Rankine et al. confirmed this result in a separate study of 11 RT patients at 3 T,^57^ which directly compared functionally guided treatment plans created using ventilation maps with those created using RBC maps.^63^ The authors assumed that the RBC signal better reflected true pulmonary function; their results showed that, on average, ventilation‐guided plans reduced the dose to high functioning areas of lung to a lesser extent than the RBC‐guided plans. The benefit of using HP ^129^Xe gas exchange MRI versus 4D CT‐derived ventilation appears to be patient‐dependent.^64^ Unlike HP ^129^Xe MRI, 4DCT is readily available and is used more commonly for functional guidance.^65^ Further study is needed to stratify patients and determine those who would benefit the most from HP ^129^Xe MRI.

Another RT application of the HP ^129^Xe gas exchange MRI technology is the quantitative imaging of radiation‐induced lung injury.^66^ A recent study of 10 RT patients reported that gas exchange metrics, both the alveolar membrane uptake and capillary RBC transfer, demonstrated changes that were both quantifiable and proportional to the regional radiation dose.^67^ Furthermore, the changes in gas exchange were 2–3 times more sensitive to radiation than the changes in ventilation. This opens the door to future studies that may further our understanding of the progression of radiation‐induced lung injury, or even explore HP ^129^Xe MRI as an early safety biomarker for symptomatic radiation pneumonitis—a potentially deadly acute toxicity from thoracic RT.

### 1.7 Considerations for biology‐guided RT in clinical practice

#### 1.7.1 Yu‐feng Wang, Petra van Houdt, Sirisha Tadimalla

Quantitative imaging biomarkers (QIBs) derived from qMRI can offer insights into the biological characteristics and physiological processes of underlying benign and tumorous tissue. As described in previous sections, several studies have demonstrated potential applications of QIBs in biology‐guided RT. With the introduction of MRI‐LINACs, biology‐guided online adaptive RT has also become practically feasible, as these systems allow daily quantitative assessment of treatment response and treatment adaptation.^68^ However, implementation of QIBs in routine clinical RT requires substantial technical and clinical validation.^28^, ^69^

Bias, repeatability, and reproducibility of the measurements can be used to evaluate technical performance of a QIB. Bias of a QIB is often reported in a phantom with known reference values (e.g., ADC in a DWI phantom) or surrogates (e.g., T_1_ relaxation times for DCE‐MRI pharmacokinetic modeling). Reporting the bias of the QIB in an accessible phantom, either commercial or with published, reproducible designs,^70^ benefits the initial development study and provides a crucial reference for other institutions and future studies to reproduce the QIB. Reproducibility refers to the variation in the measurement under different conditions (e.g., interscanner or intersite differences) and can also be measured using phantoms. Reporting reproducibility, particularly in multicenter studies, helps establish performance standards that could serve as credentialing criteria for future multicenter clinical trials. Repeatability refers to the stability of the QIB when measured under the same conditions and informs the degree of variation expected in the measurement without any treatment effects. Repeatability is particularly important for longitudinal studies, where the intention is to monitor and/or adapt to treatment response. Longitudinal phantom measurements can indicate whether significant scanner‐related changes occur during the trial, which can then be accounted for during data analysis. Test–retest imaging studies in healthy or patient participants can quantify uncertainty from normal day‐to‐day variations in vivo and during scanner operation. The repeatability of the QIB informs the reliability in detecting a treatment‐related change and facilitates the development of robust QIBs for longitudinal clinical trials.

The technical performance of a QIB is affected by variations in image acquisition and processing, which can be a significant barrier for the transition of QIBs from single‐center studies to large, multicenter clinical trials. For example, there is currently no harmonization across centers for the analysis of qMRI, leading to large variations in measurements.^71^, ^72^ Consensus guidelines for a few qMRI techniques have been developed by many ongoing and past initiatives in the MRI community, such as the European Imaging Biomarkers Alliance, Quantitative Imaging Biomarkers Alliance, Quantitative Imaging Network, and the ISMRM Open Science Initiative for Perfusion Imaging.^73^, ^74^, ^75^ However, many of these efforts are directed toward development of diagnostic biomarkers. In contrast, for biology‐guided RT, QIBs are typically applied as predictive, monitoring, or response biomarkers.^76^ For example, the HEF derived from liver DCE‐MRI described previously is potentially a predictive biomarker for RILD after RT for liver cancer and thus useful for functional avoidance in RT planning. Therefore, tailored consensus guidelines to optimize the technical performance of these QIBs specifically for biology‐guided RT are needed. This is also true for QIBs developed for biology‐guided adaptive RT on MRI‐LINACs, as the design of the MRI scanner in these systems is different from diagnostic scanners.^77^ So far, the bias and repeatability of ADC, T_1_, and T_2_ mapping on MRI‐LINACs systems have been investigated.^69^ In addition, feasibility of more sophisticated techniques, such as metabolic imaging with chemical exchange saturation transfer or OE‐MRI, has also been shown.^78^, ^79^

Clinical validation of QIBs can be achieved through prospective studies linking the QIB with the relevant biological characteristic or clinical outcome. Clinical validation of several QIBs for biology‐guided RT is underway, as described in previous sections. On MRI‐LINAC systems, day‐to‐day change in tumor volume on ADC maps is being investigated as a QIB for biology‐guided adaptive RT for GBM^80^ and head and neck cancer (clinicaltrials.gov, NCT05160714). So far, most clinical evidence is based on small cohorts in single‐center settings. An important next step forward is to validate these single‐center findings in a multicenter setting, which requires knowledge of the technical performance of the QIB.

**FIGURE 2:**
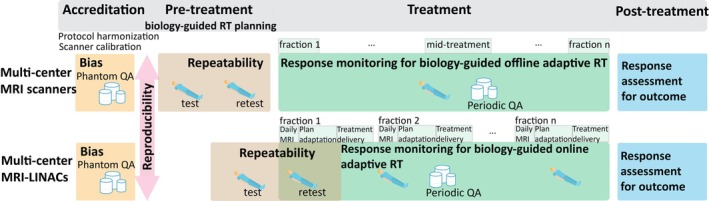
Multicenter clinical trial design framework for the development and validation of quantitative imaging biomarkers for biology‐guided radiation therapy (RT). The framework can be applied to studies using MRI scanners as well as MR‐linear accelerators (MRI‐LINACs).

Few QIBs have achieved the technical and clinical validation required for clinical translation, as these studies are often time‐consuming and costly. Nevertheless, the integration of quality assurance programs using phantoms and test–retest volunteer scans within clinical trials is feasible and can enable simultaneous technical and clinical validation of QIBs.^81^, ^82^ Figure 2 presents a framework for a multicenter clinical trial design for the development and validation of QIBs for biology‐guided RT using standalone MRI scanners as well as MRI‐LINACs. In this framework, phantom scans are used during a pretrial accreditation phase to estimate bias and reproducibility of the QIB. During this phase, harmonized protocols can be developed and tested, and any calibration factors accounting for reproducibility errors can be determined. Test–retest scans in the pretreatment phase are used to assess repeatability of the QIB. In the case of trials using MRI‐LINACs, the daily MRI scan acquired at the first treatment visit can be used as the retest measurement if acquired before the dose delivery. Reproducibility of the QIB in vivo can also be estimated in this phase by pooling data across centers, as the interscanner variability is already known from the phantom measurements. Longitudinal MRI scans performed at different treatment fractions can be used to monitor response during treatment, providing evidence for offline or online biology‐guided RT adaptation. Phantom scans performed at identical time points can be used to ensure scanner stability over the duration of the clinical trial. Finally, further MRI scans can be performed after treatment to assess response and identify correlations with treatment outcomes.

## 2 CONCLUSION

The 2024 ISMRM member‐initiated session on biology‐guided RT using MRI highlighted major advances in the clinical application of various qMRI techniques to improve RT dose planning and delivery. Beyond the more common applications of qMRI for improved accuracy of target delineation and response assessment, the presentations at this session demonstrated the rapid growth in current clinical research on identification of high‐risk, infiltrative GBM regions for focal dose escalation using spectroscopic MRI, detection of hypoxic tumor subregions using OE‐MRI to predict radiation resistance, real‐time multiparametric relaxometry for online adaptive RT with 4D‐MRF, and functional sparing of normal tissues in liver and lung RT using DCE‐MRI and ^129^Xe‐MRI, respectively. These studies, although not exhaustive, showcase recent qMRI applications for biology‐guided RT. Some techniques, like ^129^Xe‐MRI for lung RT, are site‐specific, whereas others, such as OE‐MRI for hypoxia, may have broader applicability. Translating methods from standalone MRI scanners, particularly at 3 T, to MRI‐LINACs—typically operating at lower field strengths with limited hardware capabilities—remains challenging. Biology‐guided RT on MRI‐LINACs is inherently cost‐effective, and qMRI techniques can be implemented with minimal workflow impact or patient burden. However, not all techniques are intended for on‐board imaging. For instance, spectroscopic MRI for GBM RT planning is performed days earlier on a standard scanner. Translation of any technique to MRI‐LINACs also depends on measurement complexity, postprocessing computational demands, and treatment fractionation.

MRI in the RT community is attentive to the technical challenges associated with clinical translation of these new techniques and technologies. There is a strong understanding of the imaging biomarker development pathway as well as recognition of the need for consensus. Although significant work remains, current progress in developing consensus is built on efforts on standardization of DWI—one of the earliest qMRI techniques applied in biology‐guided RT. As the most established method, DWI benefits from extensive clinical experience, but like all emerging techniques, still requires rigorous validation. As technology evolves on both standalone MRI scanners and MRI‐LINACs, widespread adoption of these techniques may become possible. This member‐initiated session was successful in fostering collaborations and discussions among international experts in the application of qMRI for biology‐guided RT. Collaboration between clinicians and industry is essential for validation and consensus on individual techniques. Future research in this field can include accelerated harmonization of techniques between standalone MRI scanners and MRI‐LINACs across field strengths and hardware, advancing artificial intelligence for fast and accurate image acquisition, reconstruction and processing, and initiating multicenter clinical trials.
